# Ambulatory high-dose methotrexate administration as central nervous system prophylaxis in patients with aggressive lymphoma

**DOI:** 10.1007/s00277-020-04341-7

**Published:** 2021-02-19

**Authors:** S. Bernard, L. Hachon, J. F. Diasonama, C. Madaoui, L. Aguinaga, E. Miekoutima, H. Moatti, Emeline Perrial, I. Madelaine, P. Brice, Catherine Thieblemont

**Affiliations:** 1 Service d’hémato-Oncologie, Assistance publique – Hôpitaux de Paris (AP-HP)- Hôpital Saint-Louis (SLS), 1, Avenue Claude Vellefaux, 75010 Paris, France; 2 Service de Pharmacie, Assistance publique – Hôpitaux de Paris (AP-HP)- Hôpital Saint-Louis (SLS), F-75010 Paris, France; 3 Université de Paris, Paris Diderot, F-75010 Paris, France; 4 INSERM 1052/CNRS 5286, F-69003 Lyon, France

**Keywords:** (*n* = 5, < 6), High-dose methotrexate, CNS prophylaxis, Ambulatory, Outpatient, Monitoring

## Abstract

High-dose methotrexate (HD-MTX) at 3 g/m^2^ is one of the strategies for central nervous system (CNS) prophylaxis in the first-line treatment of aggressive lymphomas, especially in diffuse large B cell lymphoma patients with high-risk CNS-International Prognostic Index. The objective of our study was to retrospectively analyze the safety of 2 cycles of systemic HD-MTX administered as an ambulatory regimen. Between January 2013 and December 2016, 103 patients were carefully selected on 6 criteria, including age < 60, albumin > 34, performance status 0 or 1, normal renal and hepatic functions, good understanding of practical medical guidance, and no loss of weight. Strict procedures of HD-MTX infusion were observed including alkalinization, urine pH monitoring, and leucovorin rescue. Renal and hepatic functions were monitored at days 2 and 7. MTX clearance was not monitored. Toxicities and grades of toxicity were collected according to the NCI-CTCAE (version 4.0). Among the 103 selected patients, 92 (89%) patients successfully completed the planned 2 cycles of HD-MTX on an outpatient basis. Eleven patients completed only 1 cycle, 3 because of lymphoma progression and 8 because of toxicity including 3 grade II hepatotoxicity, 2 grade I/II renal toxicity, 1 grade III neutropenia, 1 active herpetic infection, and 1 grade III ileus reflex. Reported adverse events (AE) included 92 (84%) grade I/II and 18 (16%) grade III/IV. Grade III hepatotoxicity, mostly cytolysis, was the most frequent AE observed with 8 (8%) events. Grade III/IV hematologic toxicities concerned 9 patients with 8 grade III/IV neutropenia and 1 thrombocytopenia. Renal toxicity was rare, mild, and transient, observed with 4 (4%) grade I/II events. Ambulatory administration of HD-MTX at 3 g/m^2^ without MTX clearance monitoring is safe with strict medical guidance. It requires careful selection of patients before administration, and a renal and hepatic monitoring after the administration.

## Introduction

CNS relapse is a serious event in patient with aggressive non-Hodgkin lymphoma (NHL) and associated with poor outcomes. CNS relapses in DLBCL occurs in 1 to 31% depending on the series and risk factors studied [1, 2]. In peripheral T cell lymphoma, the risk of CNS relapse has not been extensively studied and was estimated between 2.1 and 6.4% in two large retrospective series [3, 4].

The incidence of CNS relapses in both brain parenchyma and meninges is usually observed during the first 2 years of follow-up in DLBCL [5, 6]. The best strategy for preventing CNS relapse is still a matter of debate [7], in all subtypes of non-Hodgkin’s lymphoma, in particular in DLBCL. The value of prophylactic intrathecal chemotherapy is controversial since CNS relapses occur more frequently in brain parenchyma than in meninges and may be observed in patients who have received intrathecal chemotherapy [1, 8]. More aggressive CNS prophylaxis such as systemic high-dose methotrexate (HD-MTX) at > 3 g/m^2^ seems to be the best alternative in this context [9]. This strategy has been developed in the LYSA group since 1989, after an induction regimen including 4 cycles of intensified CHOP for patients with aggressive DLBCL [10]. The validation of the CNS-IPI score by Schmitz et al. [11] in 2016 rendered possible a better identification of patients with a high risk of CNS relapses.

An exhaustive review of available data about CNS prophylaxis highlights the efficiency of HD-MTX as CNS prophylaxis at a dose superior or equal to 3 g/m^2^ [12, 13]. HD-MTX administration (usually between 3 and 8 g/m^2^) is used for a variety of pediatric and adult cancers including osteosarcoma, acute lymphoblastic leukemia, and primary or secondary CNS lymphoma.

MTX is an antimetabolite-targeting folate metabolism and penetrates through cell membranes, particularly at doses where it crosses the blood-brain barrier. MTX is mainly bound (50 to 80%) to albumin in the plasma circulation and its essentially renal clearance explains the possible occurrence of severe toxicity after high-dose administration (> 500 mg/m^2^). When patients experience delayed MTX elimination, the prolonged exposure to toxic MTX concentrations can lead to significant morbidity. All of these toxicities may lead to non-reversible adverse events and mortality [14]. Regarding renal toxicities, HD-MTX can induce an acute tubular necrosis and precipitate with its metabolite in 2–4% of cases. This is a major complication of HD-MTX that may be reduced by using an antidote, the glucarpidase or carboxypeptidase G2. An increase of more than 50% in serum creatinine 36–48 h after administration of HD-MTX is considered to be predictive of a delay in the elimination of methotrexate [15]. Non-renal toxicities include hepatic, hematological, gastrointestinal, and neurological toxicities. Although severe hepatic cytolysis is rare, a simple increase of liver enzymes is frequently observed, which is usually transient and spontaneously reversible. Superficial ulcers and mucositis can affect the entire digestive tract. Neurological complications may arise because MTX interferes with transmethylation reactions which are crucial for the production of myelin. They may be either acute, immediately after treatment (3.8 to 7.8% in pediatric ALL patients) [16, 17] (mainly leukoencephalopathy) and most of the time reversible, or delayed with neurological and progressive cognitive impairment (necrotic leukoencephalopathy). MTX may also induce hematological toxicity. A study of an elderly population treated with HD-MTX for PCNSL reported 39% grade III/IV neutropenia, 16% grade III/IV anemia, and 6% grade III/IV thrombopenia [18]. Immunoallergic pneumonia leading to pulmonary fibrosis can be observed in very rare cases [19].

Selection of patients on clinical and biological features, clinical monitoring, hydratation urine alkalinization, and leucovorin rescue are associated with an improvement of morbidity and mortality related to HD-MTX [19]. An adapted patient selection and management of systemic HD-MTX administration is required [19]. Because of the risks associated with HD-MTX, most institutions in the world still require a minimum 72-h inpatient stay for administration and monitoring of serum concentrations of MTX. These hospitalizations reduce life quality of patients, and are associated with a significant cost. Several studies, especially in pediatric populations [20–22], have provided evidence that outpatient administration of HD-MTX represents a safe modality, on the condition that home intravenous hydration is administered. Few data are available for adult lymphoma populations with the indication of CNS prophylaxis. Recently, Pampin et al. reported an outpatient administration of HD-MTX with daily hospital visits to monitor creatinine value, pH level, and methotrexate levels at 24 h, 48 h, and 72 h [23].

This urged us to report our experience of an outpatient administration of HD-MTX as CNS prophylaxis without MTX clearance monitoring, but based on a careful monitoring of renal and hepatic functions and a strict selection of patients.

The aim of this study was to retrospectively analyze the procedure of an ambulatory administration of HD-MTX for CNS prophylaxis in first-line treatment, based on the renal and hepatic monitoring in a highly selected population of patients with aggressive lymphoma.

## Patients and methods

### Population

We performed a retrospective analysis of HD-MTX administration in the outpatient clinic among patients with aggressive B cell (*n* = 98) or T cell (*n* = 5) non-Hodgkin’s lymphoma (NHL) between January 2013 and December 2016 at Saint-Louis Hospital, Paris, France. All these patients were treated as first-line treatment with CHOP or ACBVP, in association with anti-CD20 for B cell lymphoma and were eligible for HD-MTX to benefit of CNS relapses prophylaxis [11]. CNS relapse prophylaxis was administrated to all patients with aaIPI ≥ 1 in (R)-ACBVP arm of treatment and as assessed by the practitioner for patients receiving (R)-CHOP based on known risk factors.

In the (R)-CHOP group (8 patients), HD-MTX was administered 21 days after the 4th (5 patients), or 6th cycle (3 patients) of R-CHOP − 375 mg/m^2^ rituximab, 50 mg/m^2^ doxorubicin, 750 mg/m^2^ cyclophosphamide, 1.4 mg/m^2^ vincristine (up to a maximum dose of 2 mg) on day 1, and 60 mg/m^2^ prednisone on days 1–5. In the (R)-ACVBP group (95 patients), the 4 cycles consisted of an induction part, each cycle containing 375 mg/m^2^ rituximab if B cell NHL, 75 mg/m^2^ doxorubicin, and 1200 mg/m^2^ cyclophosphamide on day 1; 2 mg/m^2^ vindesine and 10 mg bleomycin on days 1 and 5; and 60 mg/m^2^ prednisone on days 1–5. CNS prophylaxis was included in the sequential consolidation part, starting 4 weeks after completion of the fourth cycle of R-ACVBP, consisting of 2 cycles of MTX (3 g/m^2^), with four subsequent cycles of rituximab (375 mg/m^2^) combined with etoposide (300 mg/m^2^) and ifosfamide (1500 mg/m^2^).

All procedures performed were in accordance with the ethical standards of the institutional and national research committee and with the 1964 Helsinki declaration and its later amendments or comparable ethical standards.

#### Selection criteria for a HD-MTX infusion in outpatient clinic

Our strategy for ambulatory HD-MTX administration was to monitor renal and hepatic functions only and not MTX clearance, as this MTX clearance is not available in the outpatient setting. We then carefully selected the patients based on 6 criteria. These 6 mandatory criteria were (1) patient younger than 60 years; (2) performance status of 0 or 1 at the HD-MTX time infusion; (3) normal renal ≥ 60 ml/min and hepatic functions in the 7 days prior to HD-MTX; (4) albumin level strictly greater than 34 g/l; (5) an absence of significant weight loss (less than10% compared to baseline); and (6) a good understanding of practical medical guidance such as oral hydratation at 2 l of alkaline water per day for 3 days, discontinuation of all drugs with potential for interaction with MTX [24], and guidelines for oral calcium folinate administration after infusion. The selection criteria for patients, in this population with a prophylactic indication, were based on the existing literature on the subject with the aim of a minimal toxicity risk [19, 25, 26].

### Treatment administration

**Fig. 1:**
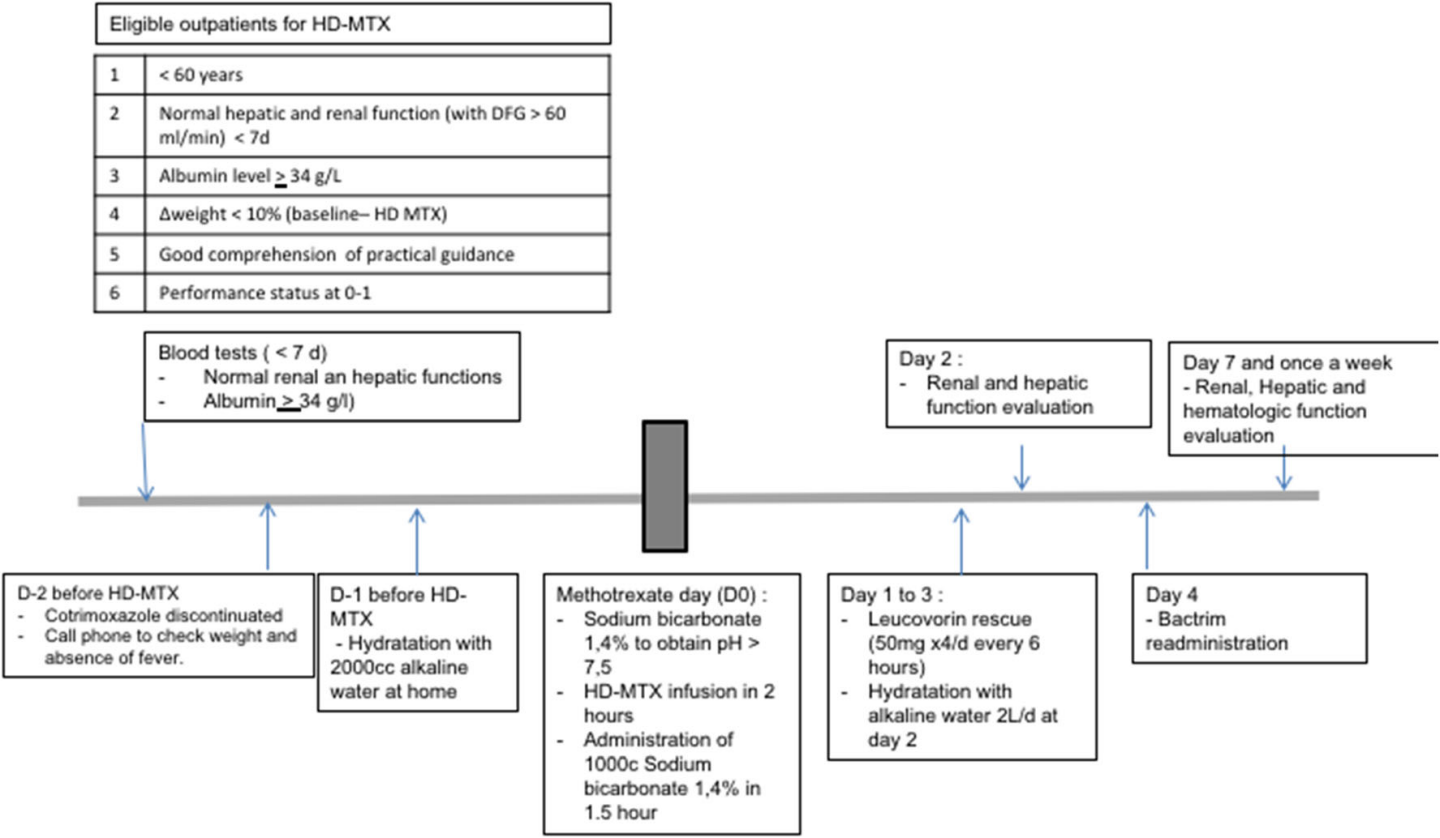
Measures associated with the administration of high-dose methotrexate on an outpatient basis

The HD-MTX infusion was managed with 3 well-defined periods: the period before infusion, the period during infusion, and the period after the infusion of the MTX. On the day before the HD-MTX infusion, patients were asked to initiate at home the urine alkalinization by drinking 2 l of alkaline water per day, as Vichy St Yorre, as well as during the 24 h following infusion. Cotrimoxazole was discontinued 2 days before HD-MTX until the end of calcium folinate administration. On the day of infusion, a 14% sodium bicarbonate solution was administered to obtain a urine pH > 7.5 within 1 h after administration. If necessary (pH > 7.5 not obtained after 1 h), we pursued alkalinization by increasing the infusion rate. Ondansetron was administered immediately before the infusion. Then, the HD-MTX was infused over a period of 2 h and followed by 1 l of 14% sodium bicarbonate over 1.5 h. After the HD-MTX infusion, the patient started the first dose of oral calcium folinate at the dose of 50 mg at H24 and pursued this treatment every 6 h during 3 days (until day 4) for a total of 12 administrations. An alkaline hyperhydratation with 2 l of alkaline water per day was also maintained during 24 h after administration. Biological analysis of creatinine clearance, ALT, AST was performed 48 h after the administration of HD-MTX and additional tests including a blood count; complete hepatic biology including ALT, AST, gamma GT, and phosphatase alkaline; and creatinine clearance was programmed once a week until the next cycle of chemotherapy. In case of increase in renal function greater than 50%, hepatotoxicity grade ≥ II, or clinical grade ≥ 2 reported toxicity (such as nausea, mucositis), the patient was contacted by the unit for inpatient hospitalization. In this procedure, MTX plasma levels were not monitored as renal and hepatic functions were precisely controlled after HD-MTX infusion (Fig. 1).

### Data collection and statistical analysis

The characteristics of the patients collected included the comorbidities and the clinical and biological characteristics at lymphoma diagnosis (sex, age, histology according to the 2016 WHO classification [27], ECOG performance status (PS), Ann Arbor stage, LDH level, extranodal sites, age-adjusted International Prognostic Index (aaIPI), CNS-IPI, induction treatment). The type of toxicities per organ (renal, hepatic, hematological, skin and mucosa, digestive) and grades of toxicities were collected according to the according to Common Terminology Criteria for Adverse Events (CTCAE 5.0), after each infusion, the first and the second infusion, of HD-MTX.

The objective of the study was to evaluate the toxicities with a HD-MTX administration in the outpatient clinic, in terms of incidence and grade per organ. All analyses were performed using Excel software.

## Results

### Characteristics of the patients

Characteristics of the 103 patients treated with HD-MTX in outpatient clinic are summarized in Table 1. The median age was 41 years old (range = 17; 60). The sex ratio was 1.4 with a larger proportion of males.

**Table 1:** Demographic and baseline characteristics of the outclinic population treated with intravenous high-dose methotrexate

	*N* = 103	Percentage
Sex M/F	60/43	58/42
Age (median)	41 (17–60)	
Histology		
- DLBCL	73	71
- PMBL	19	18
- T cell lymphoma	3	3
- Transformed follicular lymphoma	6	6
- Anaplastic lymphoma	2	2
PS		
- 0 or 1	92	89
- > 2	11	11
Ann Arbor stage		
- Stages I–II	18	17
- Stages III–IV	85	83
LDH level		
- Normal	38	37
- Upper normal	65	63
Extranodal sites		
- 0 or 1	66	64
- > 1	37	36
aaIPI		
- 0 or 1	49	48
- > 2	54	52
CNS-IPI score		
- Low risk 0 or 1	40	39
- Intermediate risk 2–3	53	51
- High risk 4–6	10	10
Induction treatment		
- R-ACBVP	89	86
- ACBVP	3	3
- Obinutuzumab-ACBVP	3	3
- R-CHOP	6	6
- CHOP	2	2

*PS*, performance status; *LDH*, lactate dehydrogenase; *aaIPI*, age-adjusted International Prognosis Index; *CNS-IPI*, central nervous system IPI

Before the initiation of HD-MTX, comorbidities and usual treatments of patients were reviewed in order to determine a potential predisposition to known toxicities. At diagnosis, 31 patients (30.1%) presented comorbidities. These included 7 arterial hypertension, 3 prior history of solid tumors (1 thyroid adenocarcinoma and 2 basal cell carcinoma), 3 type 2 diabetes (1 insulino-requiring—2 non insulino-requiring), 2 psychiatric disorders (1 anorexia and 1 depression), 2 pulmonary diseases (1 asthma and 1 sleep apnea syndrome), 3 non-active chronic viral infections (2 chronic hepatitis B, 1 chronic hepatitis C), and 11 other diseases (3 patients with glaucoma, 3 patients with hypothyroidism, 2 patients with psoriasis, 1 patient with thromboembolic disease, and 1 patient with endometriosis). Three patients presented 2 comorbidities. All the patients had normal hepatic and renal function at time of HD-MTX infusion.

A majority of patients (*n* = 80, 78% patients) did not receive any concomitant treatment. Two patients received an antidiabetic treatment, 5 an antihypertensive drug, 1 an antiviral treatment, 3 a psychiatric treatment, and 7 others (painkillers, hormonal treatments, iron, thyroid substitution). Five female patients received oral contraception.

Histological subtypes were DLBCL or transformed follicular lymphoma for 79 patients (77%), PMBL for 19 patients (18%), T cell lymphoma (NOS or ALK anaplastic lymphoma) for 5 patients (5%). At presentation most of the patients had a good performance status (*n* = 92, 89%), a disseminated stage (*n* = 85, 82.5%), and elevated LDH levels (*n* = 65, 63%). The age-adjusted IPI (aaIPI) was 2–3 for 54 patients (52%). The CNS-IPI score was retrospectively calculated. A low risk (score 0–1) was found in 40 (39%) patients, an intermediate risk (score 2–3) in 53 (51%), and a high risk (score ≥ 4) in 10 (10%) patients.

Regarding the chemotherapy induction regimen, 95 (92%) patients received an ACVBP associated with anti-CD20 for 92 patients (89%) (rituximab, *n* = 89 or obinutuzumab, *n* = 3), and 8 (8%) a CHOP associated with anti-CD20 for 6 patients. Based on Cheson criteria 2014 [28], the response assessment at the end of the standard chemotherapy induction was a complete response for 91 (88%) patients and a partial response for 12 (12%). During induction, all the patients received prophylactic antibiotics (cotrimoxazole-atovaquone in all cases, except 1 who received atovaquone alone) and antiviral treatment (valaciclovir).

### Incidence of toxicities

Among the 103 patients, 110 toxicities of any grade were reported during the 2 courses of HD-MTX chemotherapy for a total of 195 cycles. Among these toxicities, 92 (84) were grade I/II and 18 (16%) grade III/IV. Thirty-three (32%) patients presented no toxicity.

**Fig. 2:**
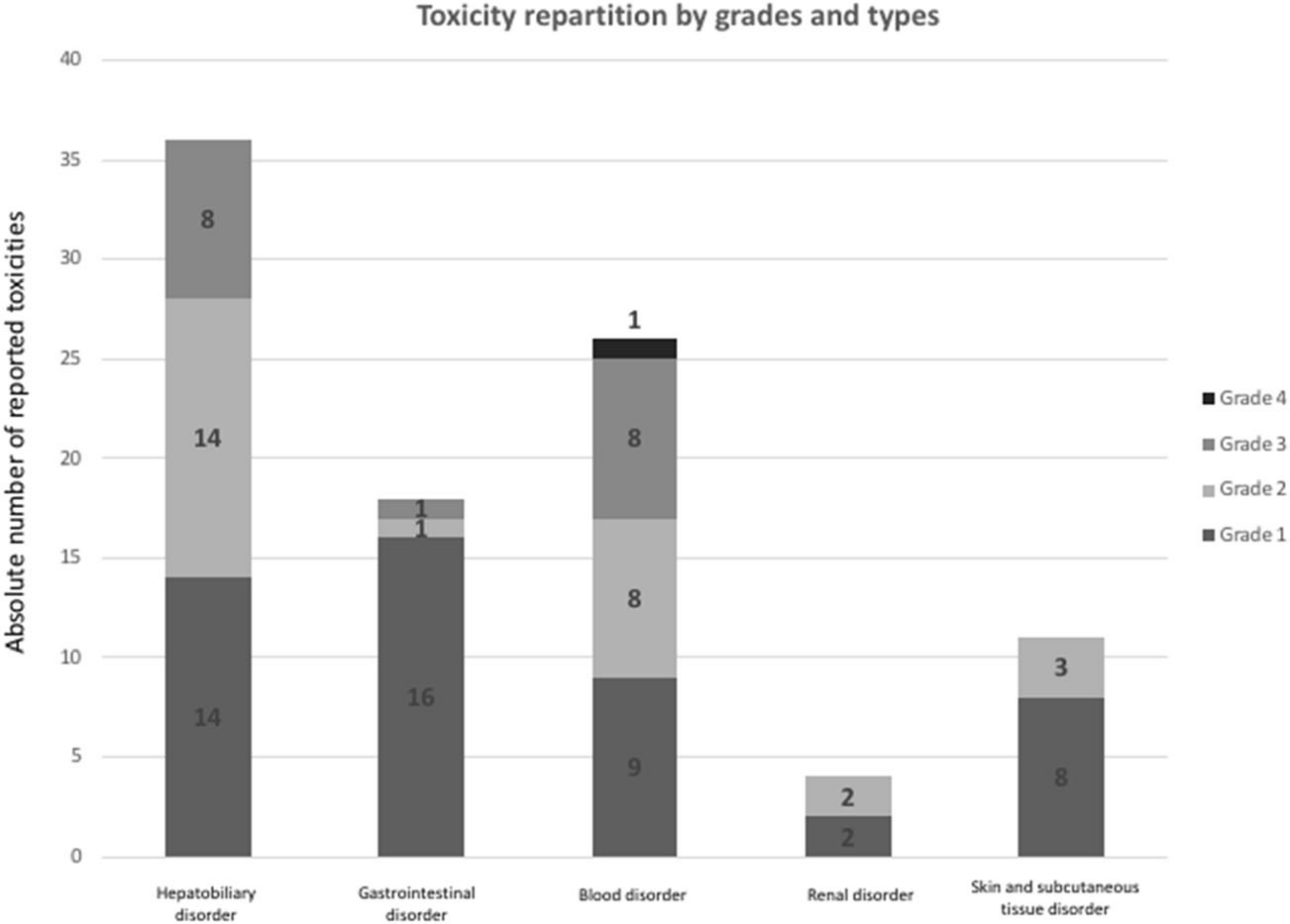
Distribution of toxicities by organs and grades among the population of 103 patients

Most of the toxicities occurred after cycle 1 (*n* = 78 toxicities, 71%), including 67 toxicities grade I/II, and 11 grade III/IV, and 32 after cycle 2 including 25 grade I/II and 7 grade III/IV (Fig. 2).

### Toxicities per organs

Hepatic toxicity was the most frequent, occurring in 36 patients (35%); this toxicity was mainly an increase in blood liver enzymes. Among them, 14 toxicities were grade I, 14 grade II, and 8 grade III. There were no grade IV hepatic toxicities. Hepatic toxicity occurred mainly after the 1st cycle (32/36, 89%). Four patients presented hepatic toxicity after both 2 cycles, including 1 that presented grade III hepatic toxicities after both. All hepatic toxicities were reversible in less than 15 days.

Skin and mucosal toxicities including mucositis and conjunctivitis (*n* = 4) occurred in 11 patients (11%) with 8 grade I toxicities, 3 grade II toxicities, and no grade III/IV toxicities, mainly after the first cycle (9/11).

Digestive toxicities mainly included nausea, vomiting, diarrhea, and constipation. Fifteen (15%) patients presented a digestive toxicity with a total of 18 events. Digestive toxicity concerned 16 grade I, 1 grade II, and 1 grade III toxicities (reflex ileus). This occurred either after cycle 1 (9/18, 50%), or after cycle 2 (9/18, 50%). Three patients presented digestive toxicities after cycle 1 and 2.

Twenty-three patients (22%) presented a hematological toxicity with a total of 26 events, either after cycle 1 (14 events) or after cycle 2 (12 events). Seventeen toxicities were grade I/II (7 neutropenia grade I/II, 8 anemia grade I/II, and 2 thrombopenia grade I/II) and 9 were grade III/IV (8 neutropenia with only 1 grade IV and 1 patient with grade III anemia). Three patients presented a cytopenia after both cycles 1 and 2 and 1 patient presented a bicytopenia after cycle 1.

Transient grade I paresthesia occurred in 9 patients (9%) mainly after cycle 1 (7/9, 78%) patients. There was no grade III/IV neurological toxicities reported.

Renal toxicity occurred in 4 patients (3.9%) and was grade I in 2 patients and grade II in 2 patients and after the first cycle for 3 of the 4 patients. There was no grade III/IV renal toxicity.

The other toxicities included arthralgia grade I (1 patient), edema grade I (1 patient), and dyspnea grade II (1 patient).

Overall, only 2 patients (2%) were hospitalized after systemic HD-MTX. The reasons were a grade III reflex ileus (3-day hospitalization) for one patient, and one because of hepatic cytolysis (24-h hospitalization).

### CNS relapses in the population

Among the 103 pts. of our study, 78 pts. remained in CR after 2 years of follow-up (76%). Five pts. in CR at the end of treatment were lost, with no follow-up. Twenty patients presented a relapse with 5 CNS relapses (4.9%) (1 was parenchymal, 1 ocular relapse, 3 leptomeningeal relapses) and 15 others relapses (15%). Three CNS relapses occur less than 6 months after the end of treatment, 1 at 1 year of follow-up, and 1 after 3 years.

### Ambulatory HD-MTX administration

All patients except for 11 (89%) received the 2 cycles of systemic HD-MTX as an ambulatory administration. Among these 11 patients, 7 received only 1 cycle of HD-MTX because of toxicities and 4 because of lymphoma progression (3 patients) and viral infection (1 patient). The toxicities that induced an arrest of treatment was a grade I/II renal toxicity in 2 patients, a cytolysis in 2 patients (grade III cytolysis for 1, grade II for 1), a grade III neutropenia in 1 patient, and a grade III digestive toxicity in 1 patient.

## Discussion

Results of our study highlighted the safety of outpatient HD-MTX administration associated with renal and hepatic monitoring only, in a highly selected population of patients aged less than 60. Overall, 89% of the patients completed the 2 cycles of HD-MTX, and 80% of the patients presented no toxicity or grade I/II toxicities. These results can be compared to other studies with patients with primary CNS lymphoma [26], or CNS prophylaxis with conventional hospitalization [29] in DLBCL patients. Other studies in pediatric osteosarcoma cohort with outpatient MTX administration showed higher level of grade III/IV toxicity, especially neutropenia (18% vs 8% in our cohort) and hepatic cytolysis (39% vs 8% in our cohort). However, the dose of HD-MTX administered in osteosarcoma is much higher, at 12 g/m^2^ in these series [30]. Only one other recent study [23] reported results on 49 de novo DLBCL outpatients receiving HD-MTX with a similar profile of toxicities. Authors reported no grade III/IV renal failure, in keeping with our observations. Also, 8% of neutropenia was reported which is in exact accordance with our study. Methotrexate serum concentrations were monitored daily starting 24 h after administration until clearance (level ≤ 0.1 μmol/l). Pampin et al. reported no MTX accumulation and no need for intensification of the rescue regimen. Our study, with a larger population (*n* = 103), supports a safe outpatient administration of HD-MTX in a highly selected population of patients, without MTX clearance monitoring, but with a very strict and careful monitoring of the renal and hepatic functions. Kidney function is a good indicator to monitor proper elimination of methotrexate [31] and should be monitored at 48 h and on day 7.

A key aspect of our work was to identify the selected population of patients eligible for an outpatient administration of H-MTX with no MTX clearance monitoring. This type of surveillance based on renal and hepatic monitoring is a novelty and requires a careful selection of the patient population. Young age less than 60, an albumin level ≥ 35 g/L, a good performance status (0–1), and a stable weight (< loss of 10% compared to baseline) are key parameters to avoid MTX clearance abnormalities [19, 32]. As usually required, a normal renal function, with a clearance ≥ 60 ml/min, is needed for a MTX clearance with no accumulation and subsequent complications. Finally, it is necessary to ensure the patient’s good understanding of practical medical guidance and compliance with the associated rules to allow the safety of outpatient treatment.

The number of any grade toxicity has been observed to be more frequent after cycle 1 than after cycle 2 of HD-MTX. This can be explained by the cumulative toxicity of prior cycles of immunochemotherapy during the induction period. A majority of patients received 4 cycles of R-ACBVP or 4 to 6 cycles of R-CHOP before the HD-MTX administration. Toxicity may be cumulative during this first cycle, especially for hematological and mucositis toxicities [10, 33]. To support this idea of cumulative toxicities after-ACVBP, Fitoussi et al. reported the toxicities after 4 cycles of R-ACVBP and highlighted the hematological (95% vs 43% grade III/IV neutropenia, 59% vs 14% anemia grade III/IV) and mucosal (30% vs 3% grade III/IV) toxicities of R-ACVBP regimen compared to R-CHOP [34, 35]. Only 11 patients did not receive the second cycle of HD-MTX. As this treatment is prophylactic, we recommended declining the second administration in case of any grade III–IV toxicity after the first infusion to avoid any cumulative toxicity.

DLBCL is a very aggressive lymphoma and most patients had a poor quality of life during the treatment, often related to repeated hospitalizations. Thanks to outpatient administration of HD-MTX, patients spent more time at home with a lower impact on their social functioning. Younger patients with DLBCL presented worse quality of life scores than more elderly patients, mainly because of social functioning alterations [36, 37]. Shorter hospitalizations are expected to be a significant factor to improve patient quality of life during the period of therapy.

In conclusion, we demonstrated that HD-MTX outpatient administration based on renal and hepatic monitoring only was feasible and safe in a selected population of patients. The selection is based on very practical and simple criteria. The organization necessary for this treatment can be easily adapted to different hospital settings.
